# Supplementary material for: Multiple Factors Influence the Incubation Period of ALS Prion-like Transmission in SOD1 Transgenic Mice
Source: Viruses. 2023 Aug 26;15(9):1819. doi: 10.3390/v15091819 (PMC10534885; doi:10.3390/v15091819)

Supplemental Figure S1. – Generation of gnSOD1-G85R:YFP.loxp construct. The approach to generating the transgene construct for gnSOD1-G85R:YFP.loxp mice is diagrammed. The starting constructs were two previously described recombinant DNA plasmids termed gnHuSOD1-3.4.5 [24] and pEF.Bos-SOD1G85R:YFP [42]. See Methods for additional details.

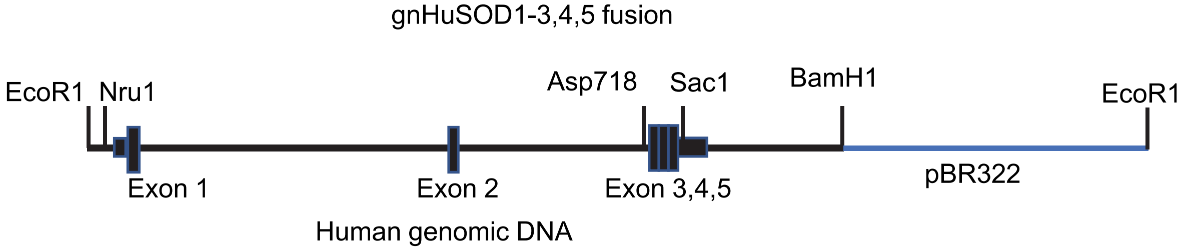

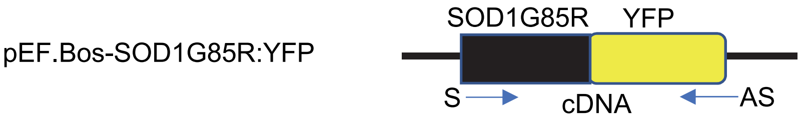

Step 1 – amplify cDNA segment in pEF.Bos-SOD1G85R:YFP with primers that introduce the loxp sequence (underlined) and unique restriction endonuclease sites that were engineered into gnHuSOD1-3,4,5.

Exon 3

Sense: 5'-gtg***ggtacc***ttaattcataatttagcttttttttcttcttcttataaatag[gctgtaccagtgcaggtcc]-3'

Antisense: 5'-ggcctcagac***gagctc***ataacttcgtataatgtatgctatacgaagttatttacttgtacagctcgtccatgc-3'

The PCR product was digested with Asp718 and Sac1 then cloned into gnHuSOD1-3,4,5 digested by Asp718 and Sac 1 using DNA ligase.

Step 2 – amplify segment in 5' end of gnHuSOD1:YFP between unique restriction sites EcoR1 and Nru1 with primers that introduce the loxp sequence (underlined)

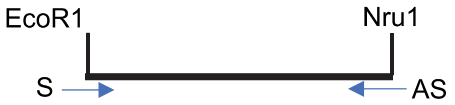

Antisense: 5'-cagcctcggg***tcgcga***gtggccgggaatg-3'

Sense: 5'-atgaccatgattac***gaattc***ataacttcgtatagcatacattatacgaagttattgccaaccaaataagaaactcta-3'

The PCR product was cloned into the construct generated in step 1, using InFusion recombination reaction, after digestion with EcoR1 and Nru1.

Step 3 – verify presence of loxp sites, G85R mutation, and YFP cDNA by DNA sequencing

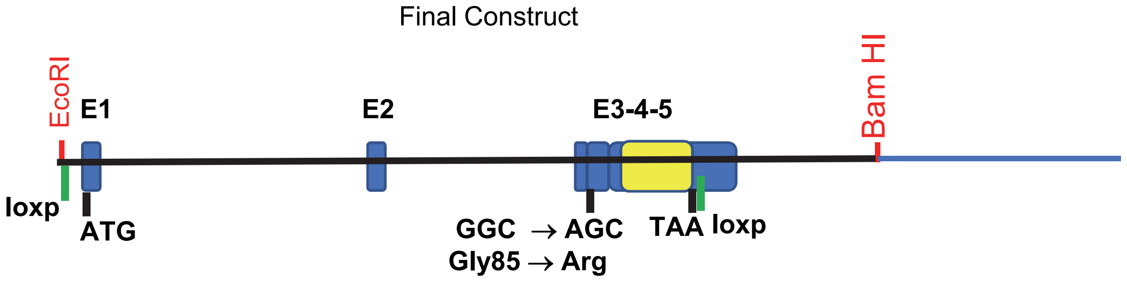

Supplemental Figure S2

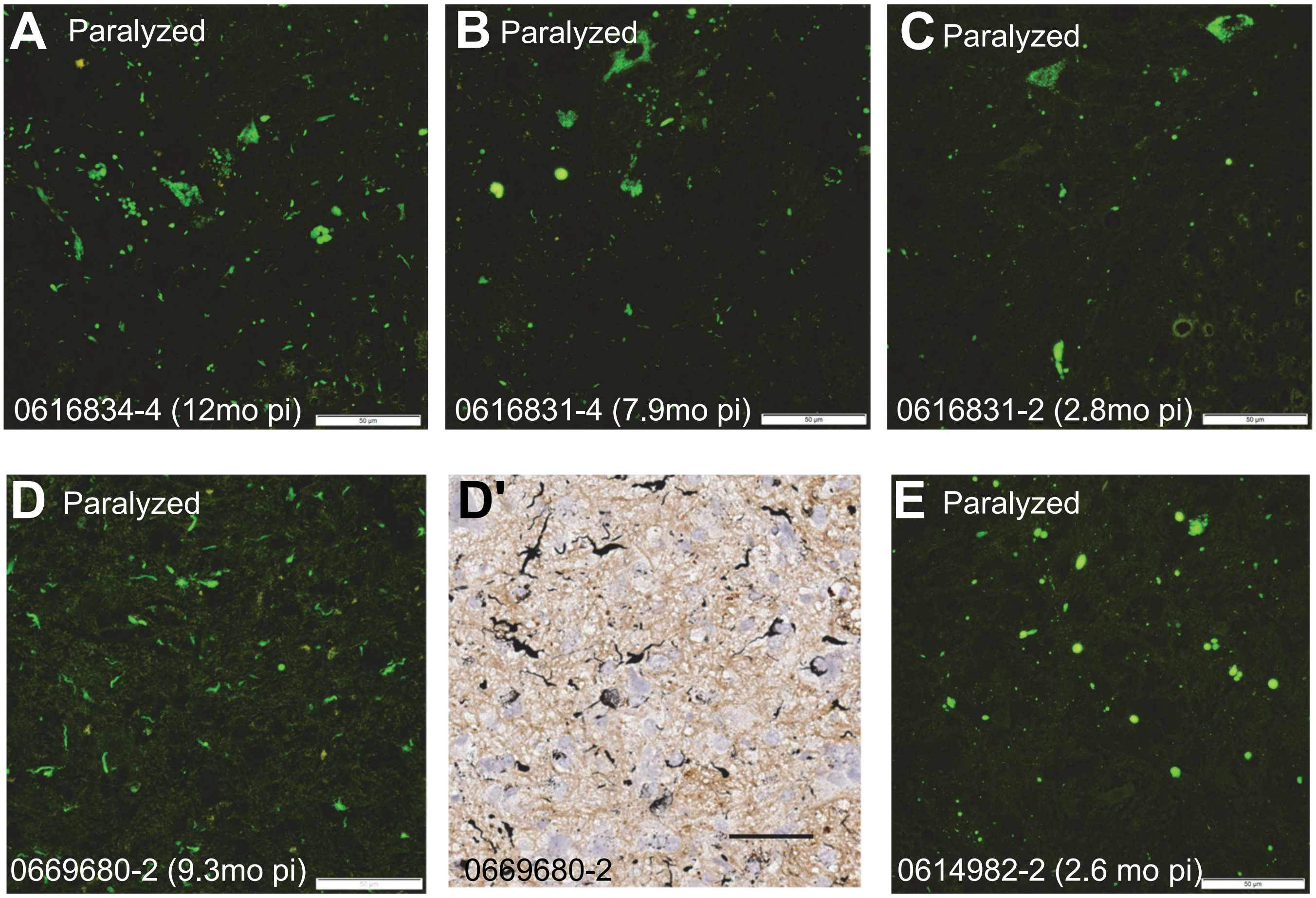

Supplemental Figure S2**.** Pathological data for animals that were used to produce inoculum. A-C) Representative images of YFP fluorescence in lumbar spinal cord from G85R-SOD1:YFP mice injected with spinal homogenate from a paralyzed G93A SOD1 mouse. Data for these animals was first described as part of cohort 3 in Ayers et al [22]. Spinal cords from these 3 animals were used to make the initial pooled inoculum. D and D') Representative images of YFP fluorescence and silver staining in lumbar spinal cord from an animal injected with fibrilized recombinant G93A SOD1. The original description of mice injected with recombinant G93A fibrils can be found in Crown et al [23]. E) Representative image of YFP fluorescence in lumbar spinal cord from an animal injected with spinal homogenate from a paralyzed G93A SOD1 mouse. This animal was part of cohort 3 that was originally described in Ayers et al [22]. Animal IDs are indicated in the bottom left of each panel. Scale bars =50μm.

Supplemental Figure S3

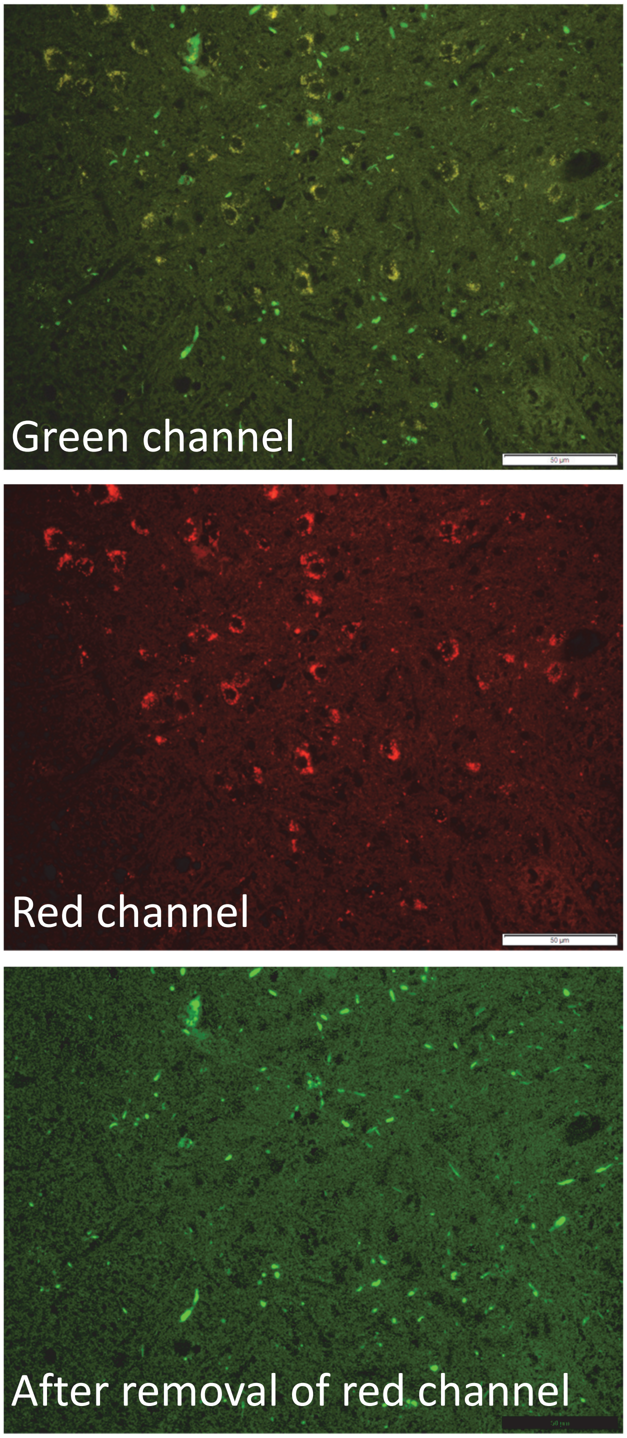

0917707-4
(20mo)

Supplemental Figure S3. Example of image processing to eliminate lipofuscin. The top panel shows the raw fluorescence image in the YFP channel. The middle panel shows lipofuscin that is visible in the red channel. The lower panel shows the processed image in which Image J was used to remove any signal that overlapped with the red channel. Scale bars = 50μm.

Supplemental Figure S4

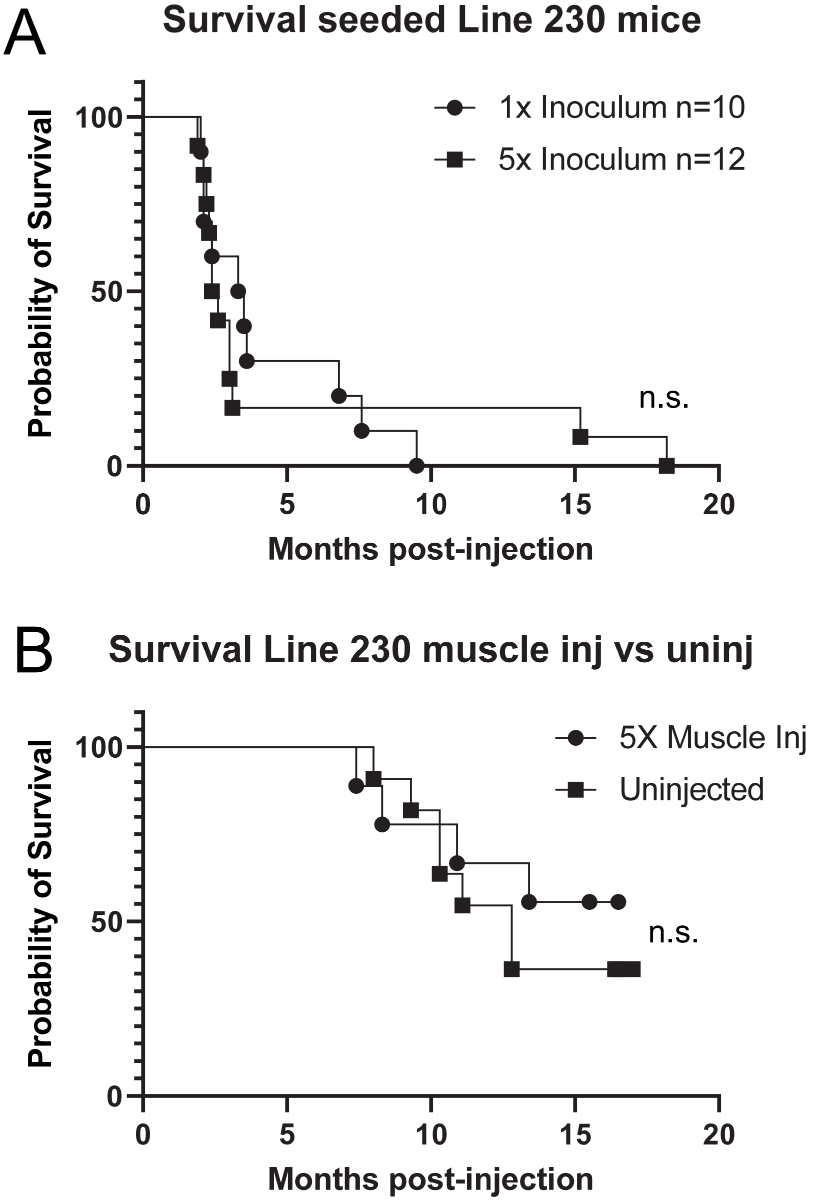

Supplemental Figure S4. Survival plots for Line 230 loxpG85R-SOD1:YFP mice. A and B) Comparison of survival in Line 230 mice injected with pooled seeding inoculum (second pool) containing second passage G93A ALS conformers. Mice were injected in sciatic nerve (A) or hindlimb muscle (B) with seeding inoculum. In each case, one cohort was injected with the seeding inoculum that had been concentrated by high speed centrifugation and resuspension in 5-fold less volume. Survival plots were generated in GraphPad Prism (version 9.5.1).

Supplemental Figure S5

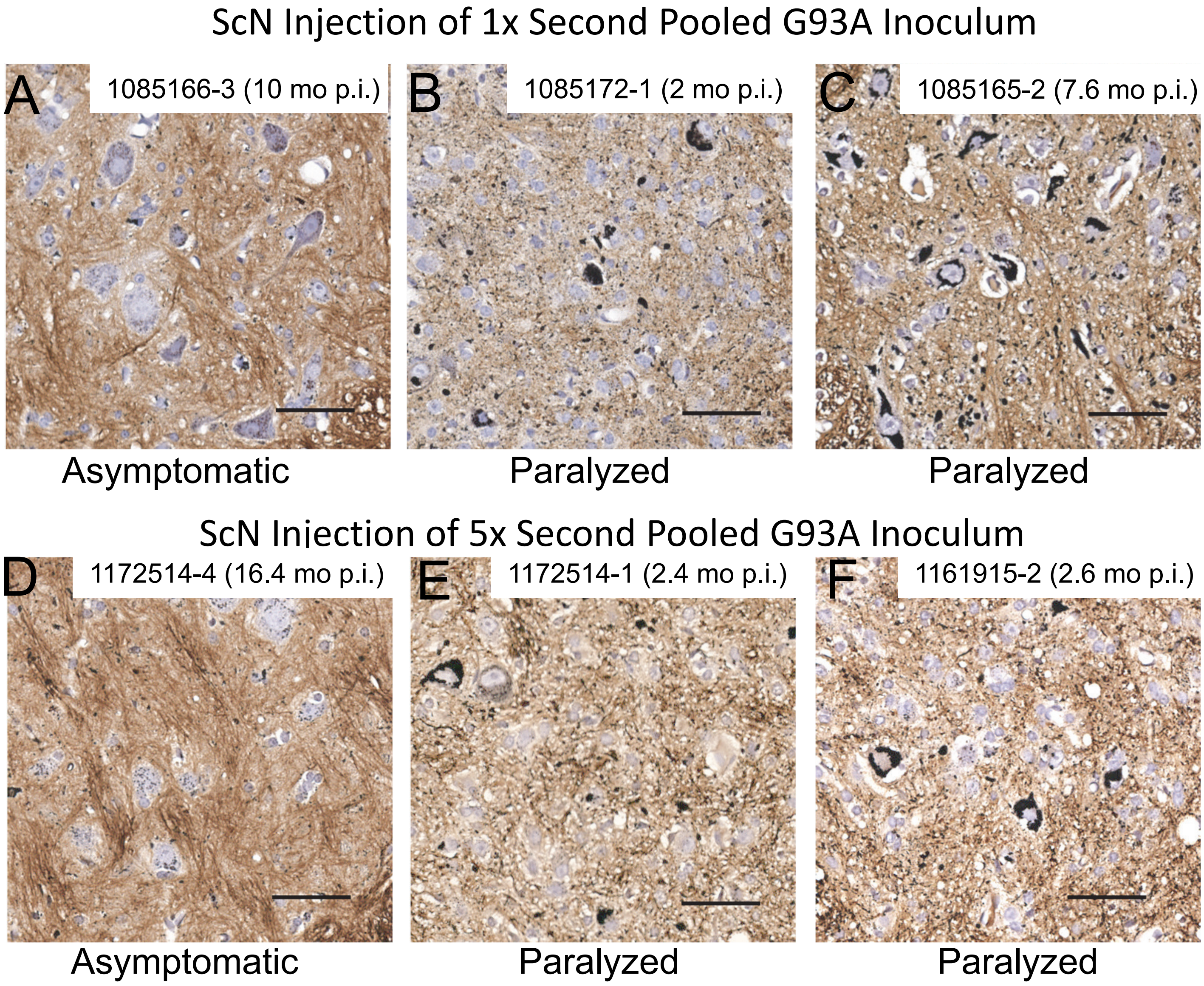

Supplemental Figure S5. Campbell-Switzer silver stains of lumbar spinal cord from Line 230 mice injected with pooled second-passage G93A seeding inoculum. A-F) Representative images from asymptomatic (A and D) and paralyzed animals (B,C,E, and F) are shown. These animals are the same as shown in Fig. 6 of the main text. Animal IDs are indicated in the top of each panel. Scale bars = 50μm.

Supplemental Figure S6

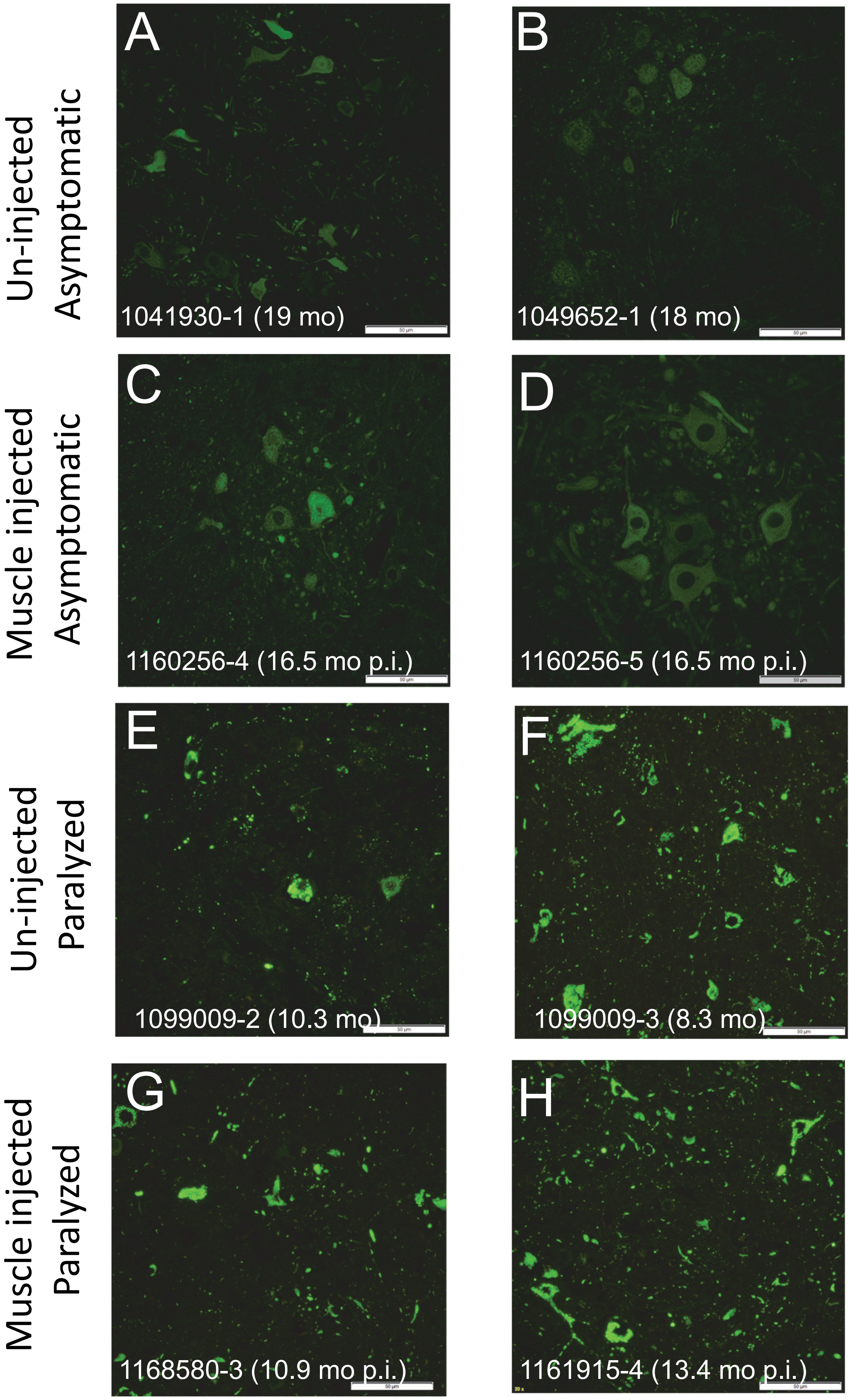

Supplemental Figuref S6. Pathological data for un-injected and muscle-injected Line 230 loxpG85R-SOD1:YFP mice. The images shown are YFP fluorescence from lumbar spinal sections. A-D) Representative images from asymptomatic mice there were either un-injected (A and B), or muscle-injected (C and D). E-H) Representative images from paralyzed mice that were either un-injected (E and F), or muscle-injected (G and H). Animal IDs are indicated in the bottom left of each panel. Scale bars =50μm.

Supplemental Figure S7. Campbell-Switzer silver staining un-injected and muscle-injected Line 230 loxpG85R-SOD1:YFP mice. The images shown are lumbar spinal sections from the same animals as shown in Fig. S6. Animal IDs are indicated in the top of each panel. Scale bars =50μm.

Supplemental Figure S7

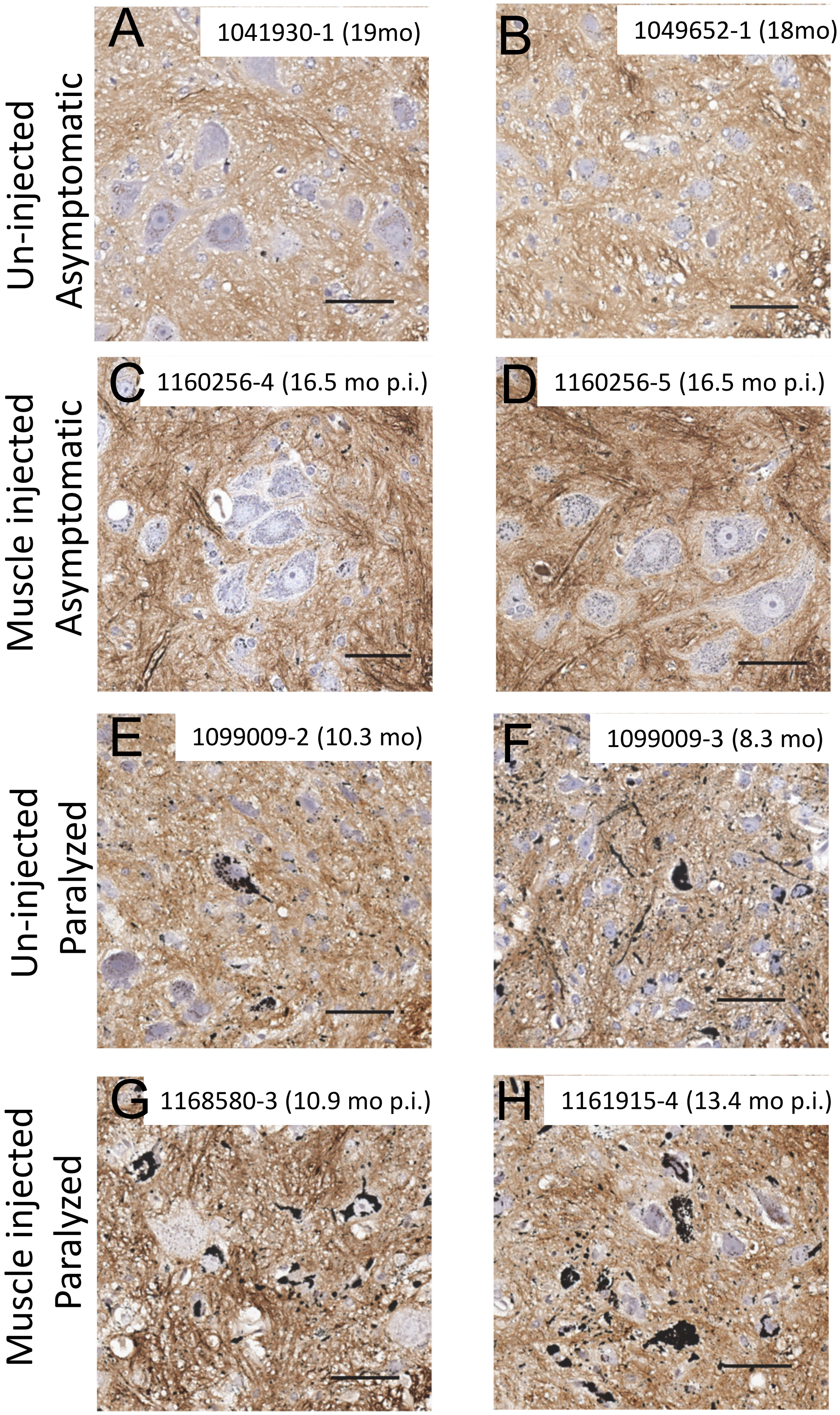

Supplement: Supplementary file 1 [file viruses-15-01819-s001.zip › viruses-2549200-supplementary.pdf]
